# P04 A vexing case of polymyalgia rheumatica

**DOI:** 10.1093/rap/rkae117.035

**Published:** 2024-11-05

**Authors:** Victoria Morrish, Nwe NiSoe, Ahmed Mohamed, Alexa Escudero, Gordon MacDonald

**Affiliations:** Royal Berkshire Hospital, Reading, United Kingdom; Royal Berkshire Hospital, Reading, United Kingdom; Royal Berkshire Hospital, Reading, United Kingdom; Royal Berkshire Hospital, Reading, United Kingdom; Royal Berkshire Hospital, Reading, United Kingdom

## Abstract

**Introduction:**

VEXAS (Vacuoles, E1 enzyme, X-linked, Autoinflammatory, Somatic) syndrome is a recently described autoinflammatory condition characterised by somatic mutations in the UBA1 gene, resulting in significant morbidity. Disease presentation can mimic several rheumatological conditions, delaying diagnosis and appropriate management. This case report presents the clinical course of a patient with a complex history of musculoskeletal symptoms and systemic inflammation, first diagnosed with polymyalgia rheumatica (PMR), who was later identified as having VEXAS syndrome almost six years after first referral to the rheumatology clinic. It illustrates the importance of considering rare autoinflammatory conditions in patients with atypical presentations of common rheumatological disorders.

**Case description:**

A 76-year-old gentleman was referred from the haematology clinic where he had undergone investigation for pancytopenia and raised inflammatory markers. Initial investigations under the haematology team revealed normal CT chest, abdomen and pelvis, unremarkable bone marrow biopsy and cytogenetics.

He presented with muscle stiffness and proximal limb girdle pain. Additional symptoms included fatigue and a diffuse non-specific erythematous rash. There was no significant past medical history and he was previously very fit and active.

Rheumatology investigations were normal, apart from persistently elevated inflammatory markers (ESR 86 mm/h and CRP 70 mg/L). A diagnosis of polymyalgia rheumatica was made and prednisolone 15 mg was started. Although he initially had a good response to this, he continued to experience significant inflammatory and constitutional symptoms. These included small joint polyarthritis, night sweats and weight loss. He was pancytopenic with associated reduced exercise tolerance, requiring transfusions and erythropoietin. To exclude other differentials, a repeat bone marrow biopsy was performed. This revealed dysplasia likely secondary to underlying inflammation, although no clonal abnormalities or vacuoles were detected. MRI right hand showed synovitis, and he was therefore treated for myalgic-onset rheumatoid arthritis. Prednisolone dose was increased temporarily and methotrexate was added, resulting in temporary improvement of his ESR 16 mm/h and CRP 25 mg/L.

The patient continued to have ongoing constitutional symptoms and developed new ataxia. Further investigations, including MRI head, gastroscopy and Whipple’s PCR were all normal. Repeat CT chest, abdomen and pelvis and PET-CT were unremarkable.

His symptoms and inflammatory markers were responsive to steroids. Given his symptoms and macrocytosis, he was tested for ubiquitin-activating enzyme UBA1 gene which was positive, confirming the diagnosis of VEXAS syndrome. Methotrexate was stopped due to worsening cytopenia, prednisolone continued and tocilizumab started. He showed significant improvement in pancytopenia and symptoms following initiation of tocilizumab.

**Discussion:**

VEXAS is characterised by somatic mutations in the UBA1 gene; its pathogenesis leads to malfunctioning methionine-41, an enzyme that initiates ubiquitylation. This leads to increased activation of innate immune pathways and induces systemic inflammation, manifesting in a variable clinical presentation. This variability makes the diagnosis challenging and leads to a median diagnostic delay of 5.5 years, similar to our patient’s six-year delay.

Our patient was initially managed as PMR due to his clinical presentation and lack of an obvious alternative diagnosis. This misdiagnosis is not unique. Often, patients later diagnosed with VEXAS are first managed as either inflammatory conditions, notably polychondritis, vasculitis or Sweet’s syndrome, or myelodysplastic syndrome (MDS).

VEXAS is hallmarked by haematological dysfunction as much as it is by inflammatory features. In fact, our patient’s first signs included a mild cytopenia which later developed into a progressive macrocytic anaemia. This is classical of VEXAS. Other haematological manifestations include thrombotic events and MDS, with minimal response to EPO-stimulating agents as seen here.

His ongoing deterioration triggered an alternative diagnosis to be sought. VEXAS is unusual as an autoinflammatory disease: its causative mechanism is a somatic mutation acquired later in life. Its diagnosis can be confirmed by inactivation of the x-linked UBA1 gene. This case highlights the need for UBA1 testing when dealing with an unexplained inflammatory phenotype, especially in men with cytopenia, multiorgan autoinflammation or MDS-like features.

VEXAS presents a management challenge. The term ‘haeamatoinflammatory’ has been created to reflect its unique nature, warranting input from both specialist teams as well as the wider MDT.

**Key learning points:**

• VEXAS should be considered as a differential when dealing with unexplained inflammatory symptoms, especially in men, multiorgan autoinflammation, vasculitis and/or MDS-like features.

• VEXAS is often progressive and transfusion-dependence is associated with a poorer prognosis. A macrocytic anaemia is a prominent feature of the condition.

• The absence or low proportion of vacuoles in bone marrow should not prevent us from testing for VEXAS if clinically indicated.

• No established protocols for VEXAS yet exist. Haematological management is supportive. Anti-inflammatory treatment is based on collective observed evidence, including the use of tocilizumbab (anti-IL6) and janus kinase (JAK) inhibitors. Allogenic haemopoietic stem cell transplantation has been successful in few patients and may represent an option for patients with severe disease.

